# Providing mothers with fetal heart monitors 

**DOI:** 10.2471/BLT.20.020720

**Published:** 2020-07-01

## Abstract

Enabling mothers in labour to monitor their baby’s heart is improving maternal and neonatal outcomes in Liberia. Tatum Anderson reports.

When Rebecca Molubah (not her real name) entered the busy government hospital in Gbarnga, Liberia, to give birth to her second child, she was anxious about her prospects.

Having been through the experience eight years earlier, the 32-year-old knew what it was like to give birth without any sort of pain relief. She had also heard enough childbirth stories with unhappy endings – including endings where the mother returned home without a baby or didn’t return home at all – to be concerned about her and her baby’s chances of survival.

The Liberian Ministry of Health has only recently begun to collect national data on perinatal deaths. However, a study of births in a rural Liberian referral hospital in 2010 showed that 11.8% (196 of 1656) deliveries in one year involved perinatal deaths, 143 of which were classified as stillbirths.

Molubah knew that one of the main reasons for the unhappy endings was a chronic shortage of doctors, nurses and midwives. So, when she walked into the maternity ward of the CB Dunbar Hospital she was pleased to see midwives in attendance.

Her pleasure turned to surprise when one of the midwives asked if she would be ready to monitor her baby’s heart rate.

“The midwife told me that my baby’s heartbeat was an important indicator of how well it was doing during labour and that if the rate went below 120 or above 160 beats per minute it might mean there was a problem,” Molubah says.

The midwife explained that because of their workload, it was sometimes difficult for the midwives to give fetal heart monitoring the attention it required. “She said that if I could do the job it would improve my baby’s chances, since the sooner midwives and doctors were made aware of a problem, the sooner they could act,” Molubah adds.

Molubah agreed to take on the task and was given a device called a sonicaid. About the size of a smart phone with a small ultrasound probe attachment, sonicaids allow the user to hear the baby’s heart and read the heart rate on a simple numerical display.

“The new initiative [used] task sharing with mothers.”

David Southall

The midwife spent 15 minutes showing Molubah how to use the device and demonstrated the difference between normal, fast and slow heart rates by tapping out a rhythm. She then told her to monitor her baby’s heart rate for about a minute immediately after the end of every uterine contraction and to inform a midwife if the heart rate got significantly faster or slower.

By taking responsibility for monitoring her baby’s heart rate, Molubah became part of a study which ran from July 2017 until October 2018 in two Liberian hospitals - CB Dunbar and CH Rennie, another government hospital located in the city of Kakata in the neighbouring county.

The study was funded by Maternal & Child Health Advocacy International (MCAI), a medical charity based in Scotland, which supports pregnant women and adolescent girls in some of the world’s poorest and most troubled countries.

 “MCAI had been working with Liberia’s health ministry for almost 9 years on task shifting, training midwives to perform obstetric physicians’ jobs, and nurses to perform advanced neonatal care,” explains Dr David Southall, MCAI’s Honorary Medical Director. “The new initiative was an extension of that work – using task sharing with mothers.”

The main aims of the study were to assess the feasibility of educating women in labour to monitor their unborn babies’ heart rates and to alert a midwife of any significant changes detected, and to assess whether the midwives would respond appropriately.

MCAI’s hope was that sharing the task would not only benefit mothers and their babies, but would also take some of the pressure off overworked health-care professionals.

One of those health-care professionals is Korpo Borzie, the midwife who led the CB Dunbar study.

“We often have to care for five or six women in labour at the same time,” Borzie says, adding that she must also take on the tasks that doctors do in other countries, including caesarean section and vacuum delivery. “You are supposed to monitor fetal heart rates while you may be dealing with somebody coming in with an incomplete miscarriage or someone with a retained placenta,” she says.

As a result, fetal heart monitoring is not always done according to best practice, important cues are missed, and standard obstetric interventions, such as improving the placental circulation by tilting the mother on her side, delivering supplemental oxygen and/or intravenous fluids, and accelerating delivery by vacuum or caesarean section are not provided.

Inevitably, babies who could have been helped are not. “If the change in heart rate is not picked up, when the nurse or the midwife is so busy with other emergencies, sometimes babies die. It’s very awful,” Borzie says.

While Southall was hopeful that the initiative would work, doubts remained. “Important questions included women’s willingness and ability to monitor their own baby, while in labour,” Southall says. “After all, these are women who are already in a stressful situation and without pain medication.” Another question related to how well midwives would respond to the mothers’ alerts.

As it turned out, the answers to those questions were largely positive. Of the 474 women asked to participate in the study, 461 women gave their informed consent. Of those, 431 (93%) completed the monitoring themselves – others handing over some of the task to midwives because of fatigue and or pain.

Those women who carried out the monitoring task, sometimes helped by their midwives, did extremely well.

“We often have to care for five or six women in labour at the same time.”

Korpo Borzie

“One woman successfully spotted tachycardia (a fetal heart rate above 160 beats per minute) after her second contraction,” says Southall. “Her baby was delivered using vacuum delivery, resuscitated and recovered within one minute with no brain damage.”

Another teenage mother spotted fetal bradycardia (a fetal heart rate below 100 beats per minute) after the fourteenth contraction. “After assessment by a doctor, an emergency caesarean section was done. The baby was resuscitated for 7 minutes, transferred to the neonatal intensive care unit and was discharged healthy after 8 days,” Southall says.

In all, 28 serious fetal heart rate changes were identified by the women, which were confirmed by a midwife or doctor in 26 cases. All 26 cases required obstetric interventions including caesarean section or vacuum delivery. All 26 babies were ultimately discharged home, apparently in good health.

Being able to help changed the mothers’ experience of labour and delivery. “Mothers who were able to monitor distress saw that if they hadn’t done it, maybe their baby would have died,” says Borzie. “Sometimes they are so excited about that.”

Adeyemo Abass Kola, an advanced neonatal nurse practitioner, who set up Liberia’s first neonatal intensive care unit (NICU) at CB Dunbar Hospital, resuscitated many of the newborns in the study.

“Before we had the NICU, they didn’t even resuscitate newborns,” he says. “Most of the time the babies come out and just need one or two minutes of resuscitation and then we return them to their mothers immediately.”

Kola is keen to point out that stillbirth is not the only potential consequence of perinatal asphyxia. “Many babies who survive [this] are brain damaged,” he says. “In a country like this where there are no resources for that type of baby, it is so worrying. Who will look after them?”

Liberia’s Ministry of Health and Social Welfare has been closely involved in the MCAI study and has given the go-ahead for fetal heart rate self-monitoring to expand to other rural public hospitals in the country.

Borzie has already begun introducing the technique and devices at another hospital. Southall is excited about the possibility of implementing the approach in other countries, pointing out that, according to the World Health Organization’s Global Health Observatory, in 2019, 17 out of 194 countries had fewer than five midwives and nurses per 10 000 of the population. 

Molubah listened to the beat of her baby’s heart for 12 gruelling hours. There was no change in the baby’s heart rate, but because there was no progress in labour, the decision was made to deliver the baby by caesarean section. The procedure went well, and Molubah welcomed a second child into the world. “She is very fine,” she says proudly. “And thank God, I am doing fine too.”

**Figure:**
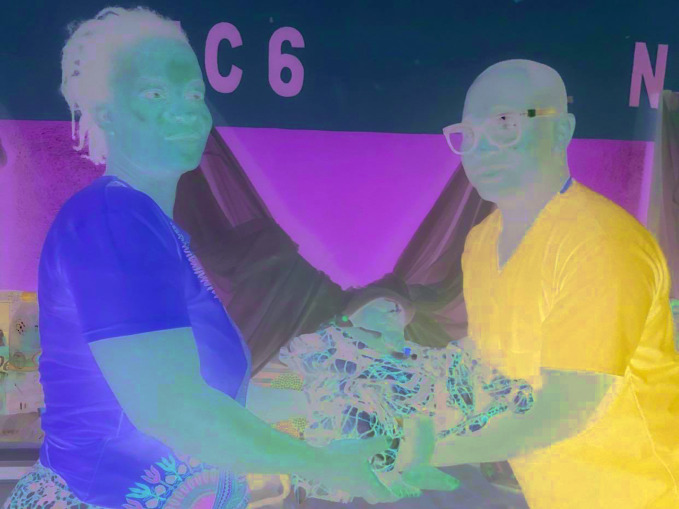
Adeyemo Abass Kola (left) with a mother and baby in the CB Dunbar Hospital neonatal unit.

Courtesy of MCAI

**Figure:**
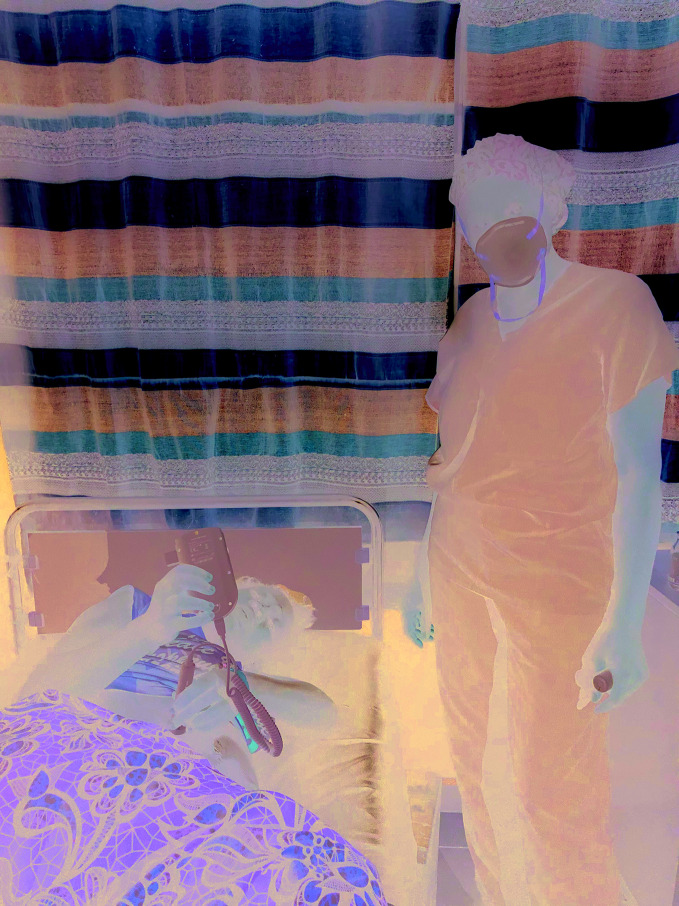
Korpo Borzie shows a mother how to monitor her baby’s heart.

Courtesy of MCAI

